# Transcriptome analysis of the hypothalamus and pituitary of turkey hens with low and high egg production

**DOI:** 10.1186/s12864-020-07075-y

**Published:** 2020-09-21

**Authors:** Kristen Brady, Hsiao-Ching Liu, Julie A. Hicks, Julie A. Long, Tom E. Porter

**Affiliations:** 1 Department of Animal and Avian Sciences, University of Maryland, 1413 Animal Sciences Building (#142), 8127 Regents Drive, College Park, MD 20742 USA; 2 Animal Biosciences and Biotechnology Laboratory, BARC, ARS, USDA, Beltsville, MD 20705 USA; 3 Department of Animal Science, North Carolina State University, Raleigh, NC 27695 USA

**Keywords:** Turkey, HPT axis, HPG axis, Gonadotropin production, Egg production

## Abstract

**Background:**

High egg producing hens (**HEPH**) show increased hypothalamic and pituitary gene expression related to hypothalamo-pituitary-gonadal (**HPG**) axis stimulation as well as increased in vitro responsiveness to gonadotropin releasing hormone (**GnRH**) stimulation in the pituitary when compared to low egg producing hens (**LEPH**). Transcriptome analysis was performed on hypothalamus and pituitary samples from LEPH and HEPH to identify novel regulators of HPG axis function.

**Results:**

In the hypothalamus and pituitary, 4644 differentially expressed genes (**DEGs**) were identified between LEPH and HEPH, with 2021 genes up-regulated in LEPH and 2623 genes up-regulated in HEPH. In LEPH, up-regulated genes showed enrichment of the hypothalamo-pituitary-thyroid (**HPT**) axis. Beta-estradiol was identified as an upstream regulator regardless of tissue. When LEPH and HEPH samples were compared, beta-estradiol was activated in HEPH in 3 of the 4 comparisons, which correlated to the number of beta-estradiol target genes up-regulated in HEPH. In in vitro pituitary cell cultures from LEPH and HEPH, thyroid hormone pretreatment negatively impacted gonadotropin subunit mRNA levels in cells from both LEPH and HEPH, with the effect being more prominent in HEPH cells. Additionally, the effect of estradiol pretreatment on gonadotropin subunit mRNA levels in HEPH cells was negative, whereas estradiol pretreatment increased gonadotropin subunit mRNA levels in LEPH cells.

**Conclusions:**

Up-regulation of the HPT axis in LEPH and upstream beta-estradiol activation in HEPH may play a role in regulating HPG axis function, and ultimately ovulation rates. Thyroid hormone and estradiol pretreatment impacted gonadotropin mRNA levels following GnRH stimulation, with the inhibitory effects of thyroid hormone more detrimental in HEPH and estradiol stimulatory effects more prominent in LEPH. Responsiveness to thyroid hormone and estradiol may be due to desensitization to thyroid hormone and estradiol in LEPH and HEPH, respectively, due to up-regulation of the HPT axis in LEPH and of the HPG axis in HEPH. Further studies will be necessary to identify possible target gene desensitization mechanisms and elicit the regulatory role of the HPT axis and beta-estradiol on ovulation rates in turkey hens.

## Background

Egg production within the turkey industry is necessary for hatching poults for meat production and has been negatively impacted by intensive selection for carcass traits. Egg production varies within commercial flocks, with low egg producing hens (**LEPH**) being more expensive per egg produced than high egg producing hens (**HEPH**). At the neuroendocrine level, egg production is regulated by the hypothalamo-pituitary-gonadal (**HPG**) axis. Proper function of the HPG axis involves feedback on the hypothalamus and pituitary by gonadal steroid hormones and can be impacted by inputs from other neuroendocrine axes, such as the hypothalamo-pituitary-thyroid (**HPT**) axis.

Within the HPG axis, feedback mechanisms of progesterone and estradiol are instrumental for follicle ovulation to occur. Progesterone feedback on the hypothalamus and pituitary triggers a preovulatory surge (**PS**) of luteinizing hormone (**LH**) and progesterone, resulting in follicle ovulation, but the role of estradiol feedback during the PS is not well characterized in the turkey hen. In the chicken, estradiol reduces gonadotropin inhibitory hormone (**GnIH**) production and exerts positive and negative feedback on gonadotropin releasing hormone (**GnRH**) production in the hypothalamus, indicating that estradiol feedback may play a role in ovulation timing [1, 2].

In addition to the HPG axis, proper function of the HPT axis is necessary for egg production to occur. The full impact of the HPT axis on reproductive function is not well understood, but studies have shown that increased activity of the HPT axis is associated with gonadal regression [3]. On the other hand, studies have shown that HPT axis activity is necessary for the initiation of egg production [4]. Additional studies examined the role of the HPT axis in the regulation of reproductive cycles in seasonally reproductive species, however, the HPT axis has not been characterized in commercial chicken or turkeys during peak egg production and has not been examined in regard to the regulation of the PS [5].

LEPH and HEPH exhibited differential expression of genes within the HPG axis, with LEPH showing higher mRNA levels for genes involved in ovulation inhibition and HEPH showing higher mRNA levels for genes involved in ovulation stimulation [6]. Furthermore, during in vitro culture of isolated pituitary cells, LEPH displayed an increased responsiveness to GnIH treatment, whereas HEPH displayed an increased responsiveness to GnRH treatment [7]. To further understand the mechanisms regulating the differential gene expression and in vitro responses seen in these two groups of hens, transcriptome analysis was performed in the hypothalamus and pituitary of LEPH and HEPH, both under basal conditions (outside of the PS) and during HPG axis stimulation (during the PS).

## Results

### Transcriptome alignment and mapping

A total of 852,343,043 sequence reads were obtained from the hypothalamus and pituitary, with an average of 35,514,293 reads per sample (Supplemental Fig. 1A). On average, 79.9% of reads mapped to the turkey reference genome (Ensembl Turkey_2.01). For each sample, read pairs were aligned with minimal discordant pairs or pairs with multiple alignments (average of 0.58 and 2.29% respectively) (Supplemental Fig. 1B). The number of reads per sample, the number of mapped reads per sample, and the number of properly aligned pairs per sample did not differ significantly between egg production or ovulatory cycle groups in either of the tissues examined.

### Overview of DEGs

**Fig. 1:**
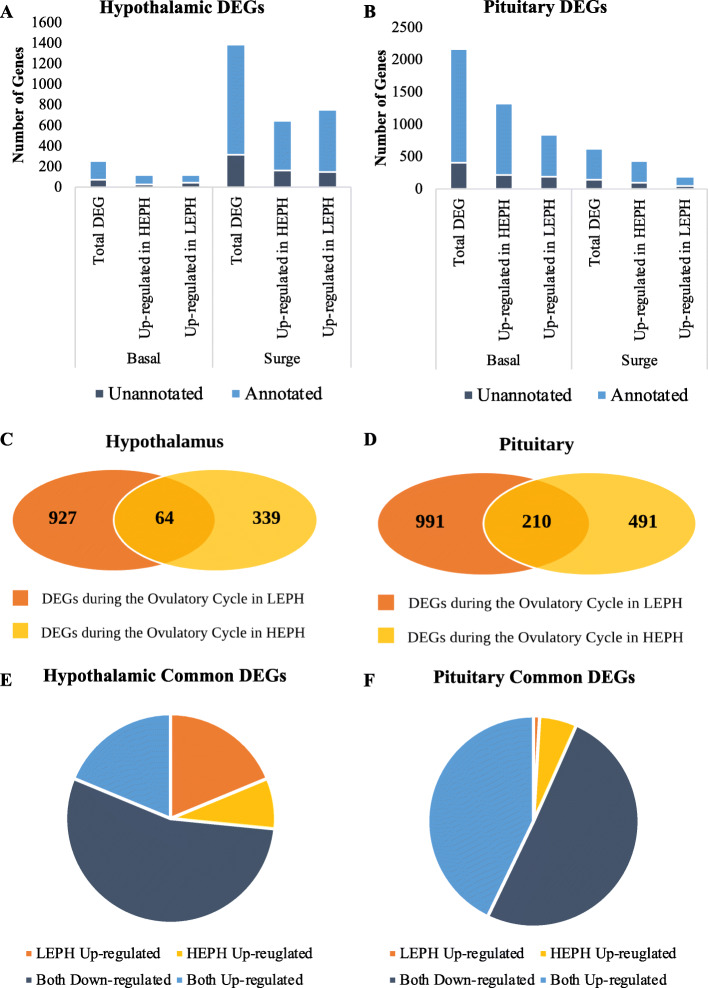
Overview of differentially expressed genes. **a** Numbers of total, up-regulated in high egg producing hens (**HEPH**), and up-regulated in low egg producing hens (**LEPH**) differentially expressed genes (**DEGs**) in the hypothalamus of LEPH and HEPH sampled outside (basal) and during (surge) the preovulatory surge (**PS**) (RPKM> 0.2, *P* < 0.05). The portion of genes that are unannotated in the turkey genome are represented in dark blue and the portion of gene that are annotated in the turkey genome are represented in light blue (Turkey 2.01, ENSEMBL annotation 98). **b** Numbers of total, up-regulated in HEPH, and up-regulated in LEPH DEGs in the pituitary of LEPH and HEPH sampled outside (basal) and during (surge) the preovulatory surge (RPKM> 0.2, P < 0.05). The portion of genes that are unannotated in the turkey genome are represented in dark blue and the portion of gene that are annotated in the turkey genome are represented in light blue (Turkey 2.01, ENSEMBL annotation 98). **c** Venn diagram showing the number of DEGs in the hypothalamus during the ovulatory cycle unique to LEPH and HEPH as well as the number of DEGs during the ovulatory cycle common to both groups of hens (RPKM> 0.2, P < 0.05). **d** Venn diagram showing the number of DEGs in the pituitary during the ovulatory cycle unique to LEPH and HEPH as well as the number of DEGs during the ovulatory cycle common to both groups of hens (RPKM> 0.2, P < 0.05). **e** Common hypothalamic DEGs during the ovulatory cycle in both LEPH and HEPH broken down by expression pattern during the PS (RPKM> 0.2, P < 0.05). **f** Common pituitary DEGs during the ovulatory cycle in both LEPH and HEPH broken down by expression pattern during the PS (RPKM> 0.2, *P* < 0.05)

A total of 1641 and 2778 DEGs was identified in the hypothalamus and pituitary, respectively (Supplemental Files 1 and 2). Analysis of the genes differentially expressed between LEPH and HEPH revealed a significantly higher number of DEGs in the hypothalamus during the PS and in the pituitary outside of the PS. In the hypothalamus, 248 DEGs were identified outside of the PS, whereas 1393 DEGs were identified during the PS (Fig. 1a). The pituitary showed the opposite trend, with 2155 DEGs outside of the PS and 623 DEGs during the PS (Fig. 1b). In the hypothalamus, equal numbers of genes were seen up-regulated in LEPH and HEPH outside of the PS, though a higher number of genes were up-regulated in LEPH during the PS. In the pituitary, both outside and during the PS, a higher number of genes were up-regulated in HEPH compared to LEPH. In the hypothalamus and pituitary, under both ovulatory cycle conditions, unannotated genes accounted for roughly 20–30% of the DEGs, indicating that further progress annotating the turkey genome may reveal additional genes involved in egg production rates or in triggering ovulation.

When comparing each hen group during the ovulatory cycle, LEPH displayed twice as many DEGs in the hypothalamus and pituitary between basal and PS conditions when compared to HEPH (Fig. 1c and d). Of the genes differentially expressed in the hypothalamus during the ovulatory cycle, unannotated genes accounted for 26% of the DEGs unique to LEPH and 47% of the DEGs unique to HEPH. Lower fractions of unannotated DEGs were seen in the pituitary during the ovulatory cycle, with unannotated genes accounting for 21% of the DEGs unique to LEPH and 27% of the DEGs unique to HEPH. In total, LEPH and HEPH shared 64 genes in the hypothalamus and 210 genes in the pituitary that were differentially expressed during the ovulatory cycle, with only four common DEGs seen in the both tissues (AKT serine/threonine kinase 3 - *AKT3*, hydroxysteroid dehydrogenase 11 beta 2 - *HSD11B2*, transgelin - *TAGLN*, and transient receptor potential cation channel subfamily C member 3 - *TRPC3*). Roughly one-fourth of the common DEGs in the hypothalamus and pituitary were unannotated as well. Of the DEGs common to both groups of hens during the ovulatory cycle, a majority showed similar expression patterns in LEPH and HEPH (73% of common DEGs in the hypothalamus and 93% of common DEGs in the pituitary) (Fig. 1e and f). A larger percentage of the common DEGs showed down-regulation in both groups of hens in the hypothalamus and pituitary compared to the percentage of DEGs that showed up-regulation in both groups of hens. A small percentage of the common DEGs showed inverse expression patterns in LEPH and HEPH (27% of common DEGs in the hypothalamus and 7% of common DEGs in the pituitary).

### Network analysis

All DEGs between LEPH and HEPH with an absolute fold change greater than 1.5 and a *P*-value less than 0.05 were submitted for Ingenuity® Pathway Analysis (**IPA**) (RPKM> 0.2) (Supplemental File 3). On average, 86.3% (± 1.36%) of submitted DEGs mapped to the IPA database. Unmapped DEGs either did not have human/mouse orthologs or suggested orthologs were not suitable to infer function (percent similarity at the protein level < 50%). Hypothalamic transcriptome differences between LEPH and HEPH included 150 genes outside of the PS and 489 genes during the PS. Pituitary transcriptome differences between LEPH and HEPH included 1577 genes outside of the PS and 301 genes during the PS. IPA analysis of the DEGs revealed two common themes in the hypothalamus and pituitary: up-regulation of the HPG axis and estradiol signaling in HEPH and up-regulation of the HPT axis in LEPH.

**Fig. 2:**
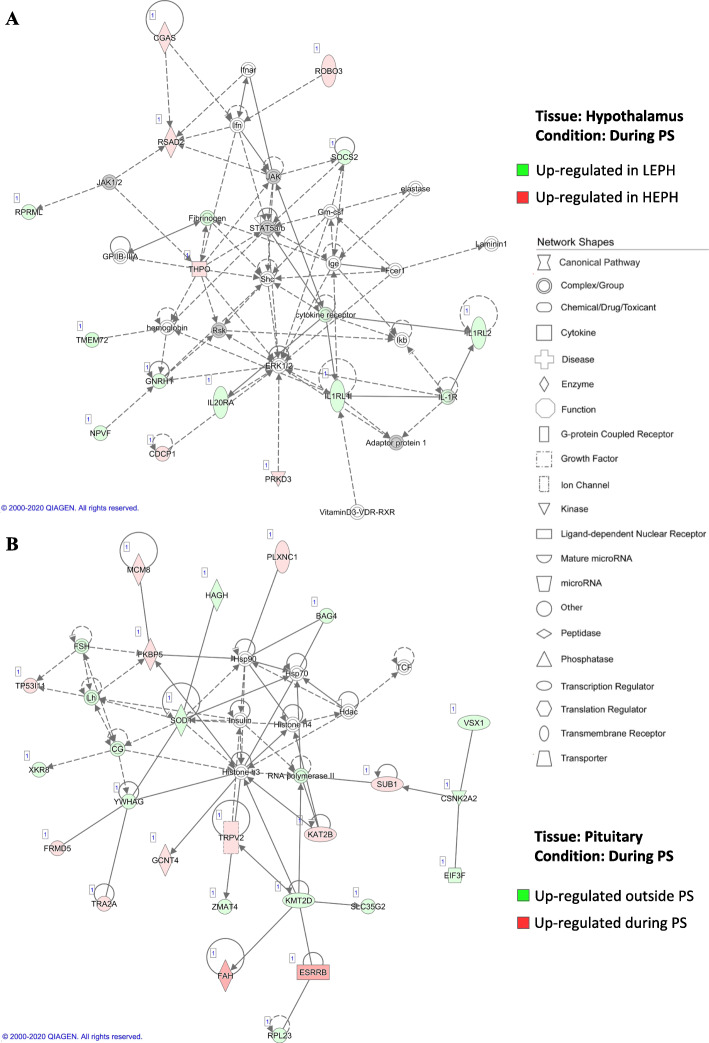
Hypothalamo-pituitary-gonadal axis networks. Ingenuity® Pathway Analysis (Qiagen, Valencia, CA) was used to generate networks to biologically interpret the expression data. Copyright permission has been obtained from QIAGEN for use of the images presented (A) Hypothalamic network comparing gene expression in low egg producing hens (**LEPH**) and high egg producing hens (**HEPH**) during the preovulatory surge (**PS**) (RPKM> 0.2, P < 0.05, |fold change| > 1.5). Green represents genes up-regulated in LEPH, whereas red represents genes up-regulated in HEPH. (B) Pituitary network comparing HEPH gene expression outside and during the PS (RPKM> 0.2, P < 0.05, |fold change| > 1.5). Green represents genes up-regulated outside of the PS, whereas red represents genes up-regulated during the PS.

Examination of the expression changes of DEGs related to the HPG axis revealed differential regulation of the HPG axis during the ovulatory cycle in LEPH and HEPH (Table 1). Outside of the PS, LEPH showed up-regulation of genes involved in prolactin signaling and androgen signaling. During the PS, LEPH showed increased inhibitory signaling related to the HPG axis, whereas HEPH showed up-regulation of estradiol and prolactin signaling. In the hypothalamus during the PS, LEPH displayed up-regulation of genes associated with ovulation inhibition as well as an abnormal up-regulation of ovulation stimulation genes when compared to HEPH (Fig. 2a). When LEPH and HEPH were compared individually outside and during the PS, LEPH displayed further increased expression of HPG axis inhibition and prolactin signaling (Supplemental Table 1). On the other hand, HEPH showed up-regulation of genes associated with estrogen signaling and follicle development during the PS in the pituitary compared to levels outside of the PS (Fig. 2b).

**Table 1:** Significant gene expression changes in the hypothalamo-pituitary-gonadal (HPG) axis between egg production levels. Fold change and significance are presented for key HPG axis genes outside and during the preovulatory surge (RPKM> 0.2, *P* < 0.05). Negative fold change values represent increased expression in low egg producing hens (**LEPH**) and positive fold change values represent increased expression in high egg producing hens (**HEPH**)

Tissue	Gene	Function	Fold	*P*-Value
**Outside Preovulatory Surge**				
Hypothalamus	PRL	prolactin signaling	−2.65	0.0245
	CYP19A1	steroid hormone biosynthesis	−1.52	0.0292
	CYP1A1	steroid hormone biosynthesis	1.92	0.0092
	HSD11B1	steroid hormone biosynthesis	1.67	0.0448
Pituitary	CGA	HPG axis signaling	1.59	0.0217
	AR	steroid hormone signaling	−1.59	0.0126
	STAR	steroid hormone biosynthesis	−16.73	0.0000
**During Preovulatory Surge**				
Tissue	Gene	Function	Fold	P-Value
Hypothalamus	GNRH1	HPG axis signaling	−2.54	0.0362
	NPVF	HPG axis signaling	−1.78	8.39E-06
	FSHR	HPG axis signaling	−7.03	0.0002
	ESR2	steroid hormone signaling	1.35	0.0218
	PRL	prolactin signaling	1.87	0.0299

**Fig. 3:**
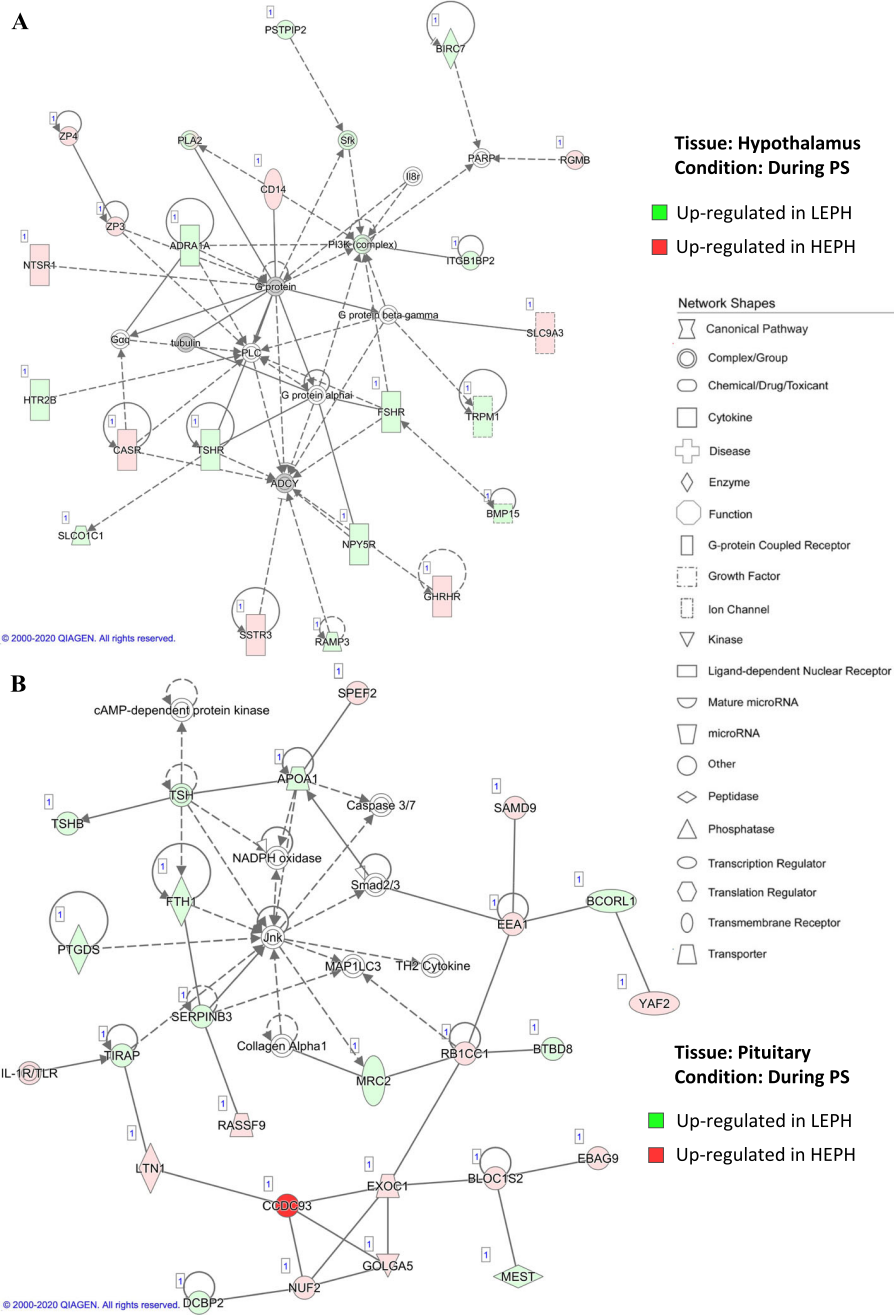
Hypothalamo-pituitary-thyroid axis networks. Ingenuity® Pathway Analysis (Qiagen, Valencia, CA) was used to generate networks to biologically interpret the expression data. Copyright permission has been obtained from QIAGEN for use of the images presented. (A) Hypothalamic network comparing gene expression in low egg producing hens (**LEPH**) and high egg producing hens (**HEPH**) during the preovulatory surge (**PS**) (RPKM> 0.2, *P* < 0.05, |fold change| > 1.5). Green represents genes up-regulated in LEPH, whereas red represents genes up-regulated in HEPH. (B) Pituitary network comparing LEPH and HEPH gene expression during the PS (RPKM> 0.2, P < 0.05, |fold change| > 1.5). Green represents genes up-regulated in LEPH, whereas red represents genes up-regulated in HEPH

DEGs up-regulated in LEPH compared to HEPH were associated with HPT axis expression in each tissue and condition examined. Examination of the expression changes of DEGs related to HPT axis revealed that LEPH exhibited up-regulation of a majority of the key genes of the HPT axis when compared to HEPH (Table 2). When LEPH and HEPH were compared individually outside and during the PS, LEPH displayed increased expression of HPT axis genes during the PS, whereas HEPH displayed decreased expression of HPT axis genes during the PS (Supplemental Table 2). During the PS, LEPH displayed higher expression of genes related to HPT axis signaling, thyroid hormone receptors, thyroid hormone transporters, thyroid hormone metabolism, and thyroid hormone synthesis when compared to HEPH in both the hypothalamus (Fig. 3a and Supplemental Fig. 2) and pituitary (Fig. 3b and Supplemental Fig. 3A)*.* Conversely, HEPH showed decreased expression of thyroid hormone transporters and genes involved in HPT axis signaling during the PS compared to levels in HEPH outside of the PS (Supplemental Fig. 3B). Generally, LEPH displayed higher expression of HPT axis genes both outside and during the PS compared to HEPH and displayed further up-regulation of the HPT axis during the PS when compared to levels outside of the PS. HEPH, on the other hand, displayed down-regulation of the HPT axis during the PS and lowered HPT axis expression both outside and during the PS when compared to LEPH.

**Table 2:** Significant gene expression changes in the hypothalamo-pituitary-thyroid (HPT) axis between egg production levels. Fold change and significance are presented for key HPT axis genes outside and during the preovulatory surge (RPKM> 0.2, P < 0.05). Negative fold change values represent increased expression in low egg producing hens (**LEPH**) and positive fold change values represent increased expression in high egg producing hens (**HEPH**)

Tissue	Gene	Function	Fold	*P*-Value
**Outside Preovulatory Surge**				
Hypothalamus	TRHR	HPT axis signaling	−1.71	0.0150
	TSHB	HPT axis signaling	−10.79	0.0368
	THRA	thyroid hormone receptor	−2.28	0.0274
	TTR	thyroid hormone transporter	3.58	0.0166
	SLO1C1	thyroid hormone transporter	−2.45	0.0197
Pituitary	CGA	HPT axis signaling	1.59	0.0217
	DIO2	thyroid hormone metabolism	−2.14	0.0012
	SLC5A5	thyroid hormone synthesis	6.48	0.0109
	ATP1B4	thyroid hormone synthesis	−3.17	0.0425
	SLC7A5	thyroid hormone transporter	−1.58	0.0088
**During Preovulatory Surge**				
Hypothalamus	TSHR	HPT axis signaling	−2.07	0.0108
	SLC5A5	thyroid hormone synthesis	3.43	0.0002
	DOUX	thyroid hormone synthesis	2.44	0.0121
	SLC26A4	thyroid hormone synthesis	−1.62	0.0186
	TTR	thyroid hormone transporter	−76.13	1.11E-15
	SLC7A5	thyroid hormone transporter	1.21	0.0359
	SLO1C1	thyroid hormone transporter	−2.83	1.34E-07
Pituitary	TSHB	HPT axis signaling	−1.76	0.0261
	DIO2	thyroid hormone metabolism	−1.61	0.0436
	TTR	thyroid hormone transporter	−11.21	0.0004

### Upstream analysis

**Fig. 4:**
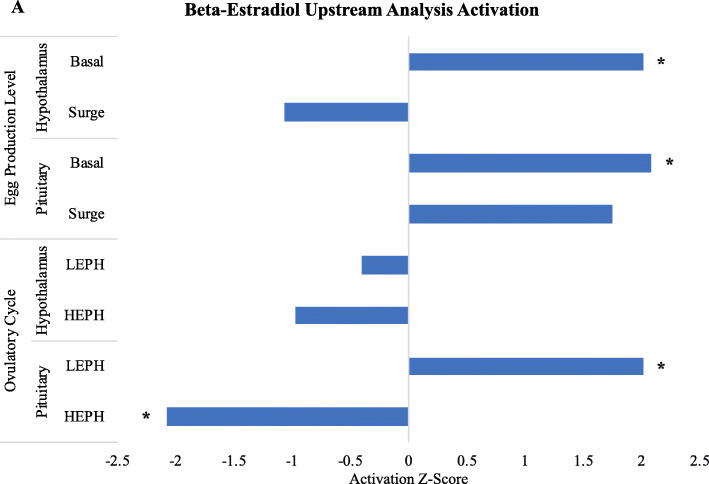
Beta-estradiol as an upstream regulator. Activation z-score calculated by Ingenuity® Pathway Analysis (Qiagen, Valencia, CA) for beta-estradiol based on differentially expressed genes (**DEGs**) (RPKM> 0.2, P < 0.05, |fold change| > 1.5). The top panel shows the calculated z-score for beta-estradiol based on DEGs between low egg producing hens (**LEPH**) and high egg producing hens (**HEPH**), both outside (basal) and during (surge) the preovulatory surge (**PS**). The bottom panel shows the calculated z-score for beta-estradiol based on DEGs between outside and during the PS in LEPH and HEPH individually. Significant predicted activation (z-score ≥ 2) or inhibition (z-score ≤ − 2) of beta-estradiol is denoted with an asterisk (*)

Analysis of the predicted upstream regulators for each comparison showed a common theme: the involvement of beta-estradiol. While the calculated Z-score varied for the comparisons examined, beta-estradiol was the only upstream regulator common to all of the comparisons (Fig. 4). Additionally, beta-estradiol was among the top five upstream regulators in the pituitary both outside and during the PS (Table 3). For the comparisons between LEPH and HEPH, beta-estradiol was significantly more active in HEPH in the hypothalamus (z-score = 2.011) and pituitary (z-score = 2.079) outside of the PS. Differentially expressed target genes of beta-estradiol in the hypothalamus outside of the PS included thyroid releasing hormone receptor (***TRHR****)*, *TSHB*, transthyretin (***TTR****)*, prolactin (***PRL***), hydroxysteroid 17 beta dehydrogenase 2 (***HSD17B2***), and aromatase (***CYP19A1***), while differentially expressed target genes of beta-estradiol in the pituitary outside of the PS included the androgen receptor (***AR***), glycoprotein hormones alpha subunit (***CGA****)*, steroidogenic acute regulatory protein (***STAR***), and solute carrier family 7 member 5 (***SLC7A5***) (Supplemental Table 3). Beta-estradiol tended to be more active in HEPH in the pituitary during the PS (z-score = 1.75), though not significantly. For the comparisons during the ovulatory cycle for each hen group, in the pituitary beta-estradiol was significantly more active during the PS for LEPH (z-score = 2.014) and significantly more active outside of the PS for HEPH (z-score = − 2.079). Differentially expressed target genes of beta-estradiol in the pituitary of LEPH included albumin (***ALB***), prolactin receptor (***PRLR***), *STAR*, and *TTR,* whereas differentially expressed target genes of beta-estradiol in the pituitary of HEPH included *CGA* and *TSHB* (Supplemental Table 4).

**Table 3:** Upstream regulators. Significant upstream regulators between low egg producing hens (**LEPH**) and high egg producing hens (**HEPH**), outside and during the preovulatory surge (RPKM> 0.2, P < 0.05, |fold change| > 1.5)

Tissue	Upstream Regulator	Molecule Type	Z-Score	*P*-Value	Target Genes
**Outside Prevoulatory Surge**					
Hypothalamus	cyclosporin A	biologic drug	0.678	1E-06	10
	MAPK8	kinase	0.889	2E-06	6
	Pkc(s)	group	0.119	3E-06	7
	FOXF2	transcription regulator	−2	5E-06	4
	FOXA2	transcription regulator	1	6E-06	7
Pituitary	DAP3	other	3	4E-08	7
	actinonin	chemical reagent	−3	1E-06	7
	ALKBH1	enzyme	2.449	5E-06	4
	NSUN3	enzyme	2.449	5E-06	4
	SIRT3	enzyme	−1.952	0.0003	8
**During Prevoulatory Surge**					
Hypothalamus	LOXL2	enzyme	−1.406	2E-06	3
	FGF2	growth factor	−0.307	3E-06	15
	beta-estradiol	chemical-endogenous	−1.064	2E-05	39
	Mek	group	1.315	2E-05	9
	BMP2	growth factor	−1.014	3E-05	8
Pituitary	ESR1	nuclear receptor	0.991	4E-06	41
	beta-estradiol	chemical-endogenous	1.749	2E-05	49
	ESR2	nuclear receptor	−0.842	0.0001	17
	CDH2	other	−1.103	0.0002	4
	cholic acid	chemical-endogenous	0.761	0.001	6

### Effect of thyroid hormone and estradiol on pituitary gonadotropin production

**Fig. 5:**
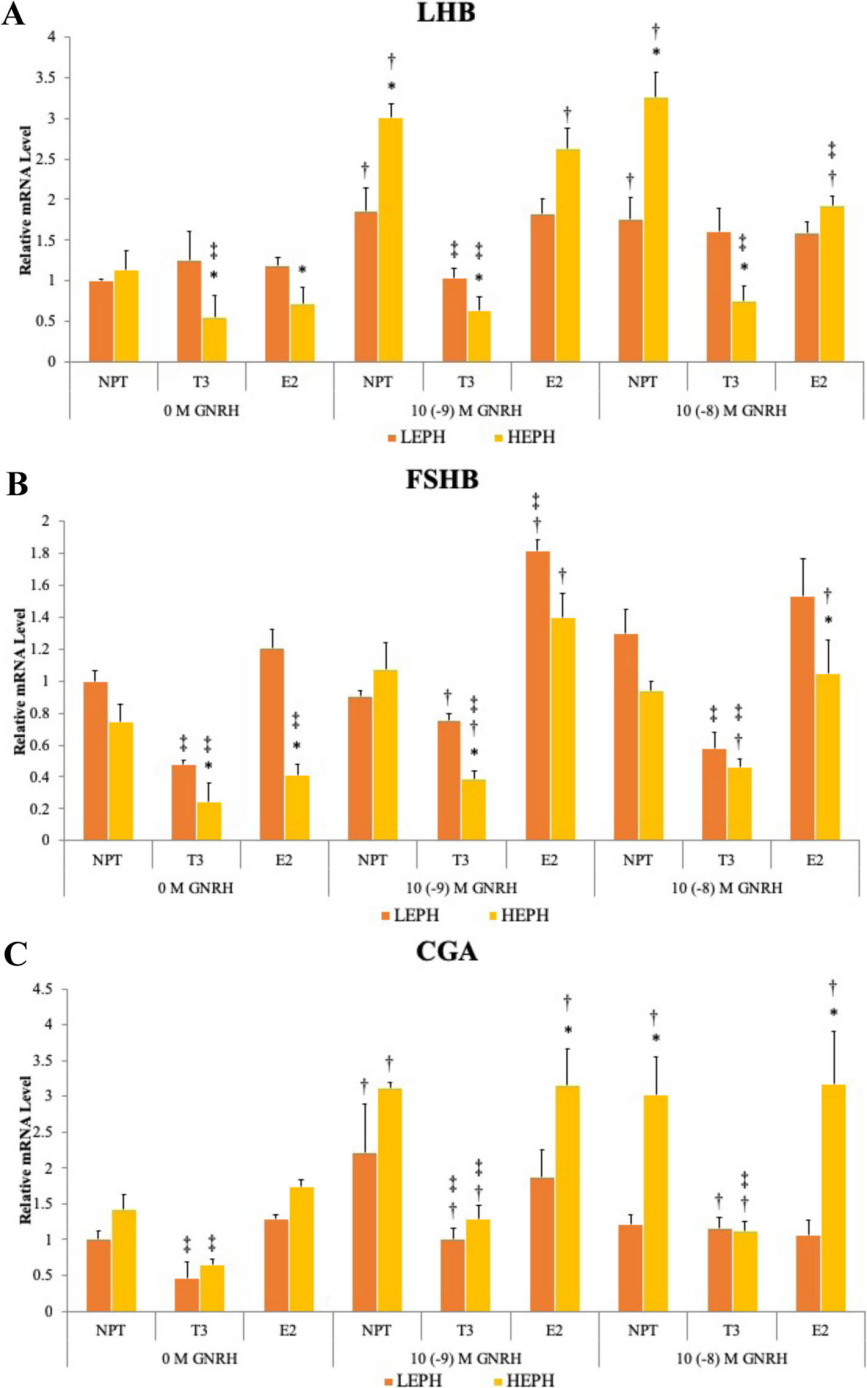
Impact of thyroid hormone and estradiol on gonadotropin production. Relative pituitary expression of the beta-subunit of luteinizing hormone (***LHB***), the beta-subunit of follicle stimulating hormone (***FSHB***), and the glycoprotein hormones alpha-subunit (***CGA***) after pretreatment with no pretreatment (**NPT**), thyroid hormone (**T**_**3**_), or estradiol (**E**_**2**_) followed by gonadotropin-releasing hormone (**GNRH**) treatment in low egg producing hens (**LEPH**) and high egg producing hens (**HEPH**). Normalized data are presented relative to LEPH basal expression for each gene. Significant expression differences between LEPH and HEPH for a given condition are denoted with an asterisk (*). Significant differences between GNRH treatments for a given egg production group are denoted with a dagger (†). Significant differences between pretreatments for a given egg production group are denoted with a double dagger (‡)

To further examine the impact of thyroid hormone and estradiol on HPG axis function, gonadotropin subunit mRNA levels were measured in pituitary cells from LEPH and HEPH after thyroid hormone pretreatment (**T**_**3**_) or estradiol pretreatment (**E**_**2**_) combined with GnRH treatment. Pituitary cells from LEPH and HEPH responded differently to each pretreatment in terms of gonadotropin subunit mRNA levels, indicating functional differences in the response of the HPG axis to thyroid hormone and estradiol that could be related to differences seen in egg production levels between the two groups of hens (Fig. 5). The in vitro effects of T_3_ and E_2_ were seen both with and without GnRH treatment, indicating that both hormones could be capable of pituitary gonadotropin regulation outside and during the PS.

T_3_ negatively impacted *LHB,* follicle stimulating hormone beta subunit (***FSHB****),* and *CGA* mRNA levels in cells from LEPH and HEPH, however the effect was more prominent in HEPH cells. T_3_ decreased *LHB*, *FSHB*, and *CGA* mRNA levels compared to no pretreatment in HEPH pituitary cells, regardless of GnRH treatment concentration. T_3_ also decreased *LHB*, *FSHB*, and *CGA* mRNA levels in LEPH pituitary cells, but only at 10^− 9^ M GnRH for *LHB*, 0 M and 10^− 8^ M GnRH for *FSHB*, and 0 M and 10^− 9^ M GnRH for *CGA*. Generally, T_3_ negatively regulated gonadotropin production, independent of GnRH treatment concentration, with a higher negative response from HEPH. These findings suggest that HEPH are more sensitive to the effect of T_3_ on gonadotropin production, whereas LEPH are more resistant to the effects of T_3_.

E_2_ decreased *LHB* mRNA levels in HEPH pituitary cells compared to no pretreatment at 10^− 8^ M GnRH. E_2_ also decreased *FSHB* mRNA levels in HEPH pituitary cells relative to no pretreatment at 0 M GnRH and increased *FSHB* mRNA levels in LEPH pituitary cells at 10^− 9^ M GnRH. E_2_ in HEPH pituitary cells decreased *FSHB* mRNA levels at lower GnRH treatment concentrations but decreased *LHB* mRNA levels at higher GnRH treatment concentrations. In contrast, E_2_ upregulated *FSHB* in pituitary cells from LEPH at 10^− 9^ M GnRH. Overall, E_2_ had varied impacts on gonadotropin production, depending on the rate of egg production of the hens.

### Confirmation of gene expression as determined by RNA sequencing

**Fig. 6:**
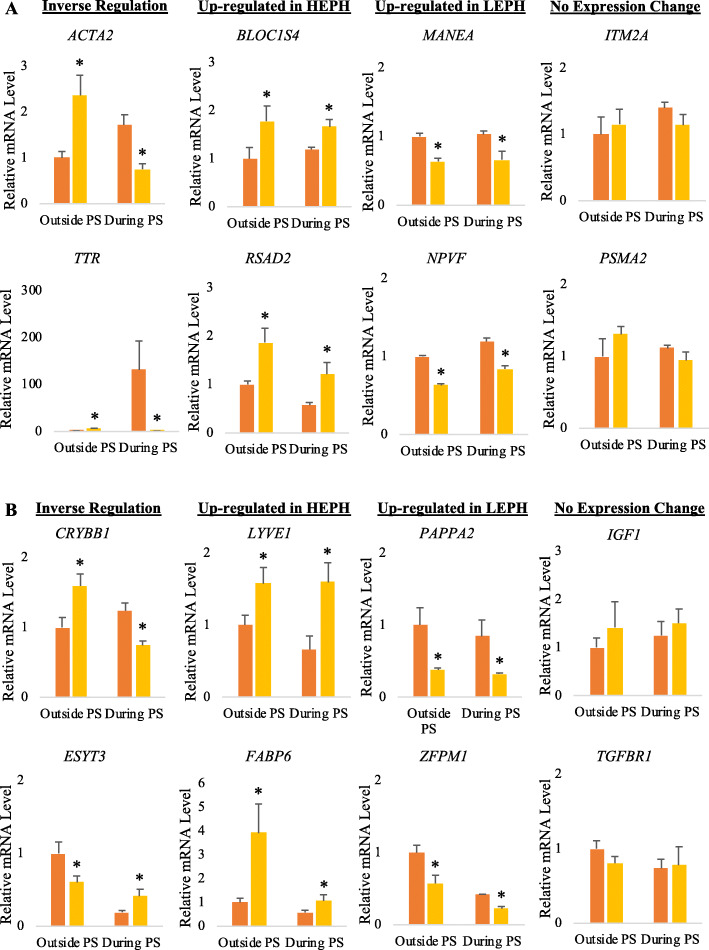
Confirmation of RNA sequencing gene expression results. (A) Confirmation by RT-qPCR of hypothalamic gene expression as determined by RNA sequencing. Six differentially expressed genes (**DEGs**), with expression patterns that showed inverse regulation in low egg producing hens (**LEPH**) and high egg producing hens (**HEPH**) outside and during the preovulatory surge (**PS**) [alpha-actin-2 (***ACTA2***) and transthyretin (***TTR***)], up-regulation in HEPH both outside and during the PS [biogenesis of lysosomal organelles complex-1, subunit 4 (***BLOC1S4***) and radical s-adenosyl methionine domain containing 2 (***RSAD2***)], and up-regulation in LEPH both outside and during the PS [mannosidase endo-alpha (***MANEA***) and neuropeptide VF precursor (***NPVF***)], as well as two genes that were not differentially expression in LEPH and HEPH either outside or during the PS [integral membrane protein 2A (***ITG2A***) and proteasome subunit alpha 2 (***PSMA2***)] were confirmed through RT-qPCR. Normalized data are presented relative to LEPH expression outside of the PS for each gene. Significant expression differences between LEPH and HEPH for a given condition are denoted with an asterisk (*). (B) Confirmation by RT-qPCR of pituitary gene expression as determined by RNA sequencing. Six DEGs, with expression patterns that showed inverse regulation in LEPH and HEPH outside and during the PS [crystallin beta B1 (***CRYBB1***) and extended synaptotagmin 3 (***ESYT3***)], up-regulation in HEPH both outside and during the PS [lymphatic vessel endothelial hyaluronan receptor 1 (***LYVE1***) and fatty acid binding protein 6 (***FABP6***)], and up-regulation in LEPH both outside and during the PS [pappalysin 2 (***PAPPA2***) and zinc finger protein, FOG family member 1 (***ZFPM1***)], as well as two genes that were not differentially expression in LEPH and HEPH either outside or during the PS [insulin like growth factor 1 (***IGF1***) and transforming growth factor beta receptor 1 (***TGFBR1***)] were confirmed through RT-qPCR. Data are presented relative to LEPH expression outside of the PS for each gene. Significant differences between LEPH and HEPH for a given condition are denoted with an asterisk (*)

Expression patterns of 8 genes per tissue were confirmed through RT-qPCR. Confirmation genes were equally distributed to have one of four expression profiles: genes showing up-regulation in HEPH compared to LEPH (both outside and during the PS), genes showing up-regulation in LEPH compared to HEPH (both outside and during the PS), genes showing up-regulation in one hen group outside of the PS and up-regulation in the other hen group during the PS, and genes showing no changes in expression between hen groups (both outside and during the PS). Each of the confirmation genes examined in the hypothalamus (Fig. 6a) and pituitary (Fig. 6b) showed expression profiles similar to those obtained through RNA sequencing.

## Discussion

Though 4419 DEGs were identified between LEPH and HEPH collectively in the hypothalamus and pituitary outside and during the PS, analysis of the gene expression changes during the PS in each egg production group provided 242 common genes showing similar expression patterns in both egg production groups. Among the genes in the hypothalamus showing similar expression patterns during the PS in both groups of hens was fatty acid 2-hydroxylase (***FA2H****)* and somatostatin (***SST***). *FA2H*, which was up-regulated in both groups of hens during the PS, is involved in myelin production, which is essential for proper nerve conduction [8]. *SST,* which was also up-regulated in both groups of hens during the PS, is the main inhibitory hormone of the somatotropic axis but has been shown to inhibit GnRH neuron activity in mice [9]. Among the genes in the pituitary showing similar expression patterns during the PS in both groups of hens was Pre-MRNA processing factor 19 (***PRPF19****)*. *PRPF19*, which was down-regulated in both groups of hens during the PS, has been shown in mouse models to impact the splicing of gonadotropin subunits [10]. Common DEGs with similar expression patterns during the ovulatory cycle in both LEPH and HEPH could indicate a potential role for these genes in the regulation of ovulation.

On the other hand, analysis of the gene expression changes during the PS in each egg production group revealed 32 common DEGs displaying inverse expression patterns in LEPH and HEPH. Of the hypothalamic common DEGs showing inverse expression patterns between LEPH and HEPH, proteasome 26S subunit, non-ATPase 2 (***PSMD2***) displayed up-regulation in HEPH and down-regulation in LEPH during the PS. In mice, mutations in *PSMD2* have been associated with decreased thyroid hormone production [11]. Of the pituitary common DEGs showing inverse expression patterns between LEPH and HEPH, NADH dehydrogenase 4 (***ND4***) and cyclooxygenase-2 (***COX2***) have been previously associated with reproductive functions [12, 13]. Both *ND4* and *COX2* showed up-regulation in HEPH and down-regulation in LEPH during the PS. Swine selected for high ovulation rates displayed higher pituitary *ND4* gene expression when compared to control lines [12]. *COX2* encodes the rate limiting enzyme in prostaglandin production, and deletion of *COX2* in mice results in decreased ovulation [13]. Common DEGs during the ovulatory cycle with inverse expression patterns in LEPH and HEPH could signify a possible role in the regulation of egg production rates.

### The HPG axis

Pathway analysis of DEGs indicated upregulation of HPG axis inhibitory pathways as well as abnormal expression of HPG axis stimulatory pathways in LEPH compared to HEPH. In the hypothalamus during the PS, LEPH exhibited up-regulation of neuropeptide VF precursor (***NPVF****),* which encodes avian gonadotropin inhibitory hormone (***GNIH***) and of gonadotropin releasing hormone 1 (***GNRH1***) (Fig. 2a). GnIH negatively regulates the HPG axis to decrease gonadotropin production in the pituitary [14]. Up-regulation of *NPVF* may play a role in reduced ovulation rates seen in LEPH. *GNRH1* mRNA levels were previously shown to decrease during the PS in hens with average egg production, whereas in the present study, LEPH showed increased expression relative to HEPH [15]. In the same study, no expression changes in *NPVF* were seen during the PS in average egg producing hens, whereas in the present study, LEPH showed up-regulation of *NPVF* [15]. Up-regulation of *GNRH1* during the PS may prevent hormone levels from returning to basal levels, prolonging the interval between ovulations.

Endocrine functions of the pituitary, in terms of reproductive hormone production and the regulatory feedback loops involved, showed differential expression between LEPH and HEPH. When comparing HEPH outside and during the PS, HEPH showed down-regulation of follicle stimulating hormone (**FSH**) and LH during the PS (Fig. 2b**)**. Decreased LH during the PS is consistent with decreased mRNA levels for the beta-subunit of LH (***LHB***) seen in average egg producing hens during the PS [15]. Additionally, in this network, casein kinase 2 alpha 2 (***CSNK2A2****)* is down-regulated in the pituitary of HEPH during the PS. *CSNK2A2* encodes an uncharacterized protein in avian species but this protein was shown to be decreased in laying geese pituitaries when compared to non-laying geese, indicating a possible role in egg production or ovulation [16]. HEPH displayed decreased expression of HPG axis stimulatory genes and increased expression of androgen and prolactin signaling. Prolactin signaling showed inverse trends in LEPH and HEPH and was impacted by the PS. Prolactin signaling has been shown to impact LH release in mammals and was up-regulated in HEPH during the PS, indicating a possible role in the shortened ovulation intervals seen in HEPH [17]. Both LEPH and HEPH showed down-regulation of gonadotropin releasing hormone receptor (***GNRHR***) during the PS, which was also seen in average egg producing turkey hens during the PS [15]. Generally, HEPH displayed down-regulation of the HPG axis during the PS, whereas LEPH displayed up-regulation of both genes that stimulate and inhibit the HPG axis during the PS, presumably leading to a longer ovulation interval in LEPH.

A potential role for estradiol signaling in the regulation of ovulation frequency was also suggested in though pathway and upstream analysis. HEPH showed up-regulation of estrogen related receptor beta (***ESRRB****)* during the PS compared to levels seen outside of the PS (Fig. 2b). Estrogen related receptors are ligand-dependent transcription factors capable of estradiol binding. Though the function of estrogen related receptors in avian reproduction have not been characterized, functional analysis of estrogen related receptors in knock-out mice and zebrafish models indicate that estrogen related receptors are essential for female reproduction [18]. Under in vitro culture conditions, estradiol treatment impacted gonadotropin subunit mRNA levels (Fig. 5). Previous studies in chickens have shown estradiol to inhibit pituitary LH production, as was seen in pituitary cells from HEPH in the current study [19]. The effect of estradiol on *FSHB* mRNA levels has not been examined in avian species but estradiol injections in quail did not impact FSH plasma levels, which is consistent with the mRNA levels seen in cells from HEPH following estradiol treatment [20]. Additionally, the predicted involvement of beta-estradiol as an upstream regulator with target genes involved in the HPG and HPT axes across all conditions examined supports the hypothesis that beta-estradiol feedback on the hypothalamus and pituitary impacts the ovulatory process and possibly egg production rates.

### The HPT axis

Pathway analysis of DEGs also indicated upregulation of HPT axis in LEPH compared to HEPH. In the hypothalamus during the PS, LEPH displayed increased expression of thyroid stimulating hormone receptor (***TSHR***) and solute carrier organic anion transporter family member 1C1 (***SLCO1C1***) relative to HEPH (Fig. 3a). In the pituitary during the PS, LEPH displayed increased expression of the beta-subunit of thyroid stimulating hormone (***TSHB***) in contrast to HEPH (Fig. 3b). *TSHR* expression in the hypothalamus is related to short loop feedback control on thyrotropin releasing hormone signaling [21]. Retrograde regulation of *TSHB* on the hypothalamus has also been implicated in increased GnRH production in response to a changing photoperiod in seasonally reproductive birds [22]. It is plausible that retrograde *TSHB* feedback on the hypothalamus could also be involved in the timing of ovulation, due to the role of *TSHB* in GnRH signaling initiation coupled with the finding that clock genes impact *TSHB* pituitary expression in several mammalian species [23]. *SLCO1C1* is a thyroid hormone transporter that participates in transporting thyroid hormone across the blood-brain barrier [24]. Up-regulation of *SLCO1C1* in LEPH during the PS would allow greater thyroid hormone concentrations in the hypothalamus, which could ultimately have genomic effects on ovulation rates [25]. Additionally, in the hypothalamus during the PS, LEPH showed up-regulation of solute carrier family 16 member 12 (***SLC16A12***) and integrin (encoded by ***ITGAV*** and ***ITGB3***) relative to HEPH (Supplemental Figs. 2A and B). *SLC16A12* encodes a thyroid hormone transporter similar to *SLCO1C1,* allowing greater transport of thyroid hormone past the blood brain barrier in LEPH rather than HEPH [24]. Integrin is a plasma membrane receptor capable of binding thyroid hormones to elicit non-genomic actions of thyroid hormone, such as protein translocation and phosphorylation [25]. Up-regulation of integrin in the hypothalamus of LEPH relative to HEPH during the PS, infers possible non-genomic implications of thyroid hormone through the binding of integrin receptors located on the plasma membrane of hypothalamic cells in LEPH [26].

In the pituitary during the PS, HEPH showed up-regulation of iodothyronine deiodinase 1 (***DIO1***) relative to LEPH (Supplemental Fig. 3A). *DIO1* is capable of converting thyroid hormone to the biologically active form but is also capable of thyroid hormone deactivation [27]. Increased thyroid hormone deactivation could mitigate the effect of thyroid hormone on the tissues of the HPG axis in HEPH. When comparing HEPH outside and during the PS, HEPH showed down-regulation of *TSHB* in the pituitary during the PS (Supplemental Fig. 3B). Thyroid stimulating hormone (**TSH**) acts on the thyroid gland to promote the synthesis of thyroid hormones [28]. Down-regulation of *TSHB* during the PS in HEPH could indicate lower circulating levels of TSH, ultimately impacting circulating thyroid hormones. Furthermore, under in vitro culture conditions, thyroid hormone treatment impacted gonadotropin subunit mRNA levels in the pituitary (Fig. 5). The negative impact of thyroid hormone was seen in pituitary cells from both LEPH and HEPH, through the impact was more substantial in HEPH cells. Negative regulation of *LHB*, *FSHB*, and *CGA* by thyroid hormone treatment was also reported in male rats [29, 30]. One possible mechanism for response differences to T_3_ between LEPH and HEPH is desensitization or down-regulation of thyroid hormone receptors in LEPH due to the general up-regulation of the HPT axis seen in the hypothalamus and pituitary of LEPH. Thyroid hormone receptor desensitization in the liver has been documented after thyroid hormone injections in mice and in vitro thyroid hormone treatment decreased thyroid hormone receptor expression in rat pituitary cells [31, 32].

## Conclusions

Hypothalamic and pituitary transcriptome analysis of LEPH and HEPH provided insight into the involvement of the HPT axis and estradiol signaling in the regulation of egg production rates. LEPH displayed higher expression of genes related to the HPT axis when compared to HEPH. During the PS, LEPH further up-regulated the HPT axis in contrast to HEPH. Beta-estradiol was activated as an upstream regulator in tissues from HEPH compared to LEPH under basal conditions. Timing of beta-estradiol activation relative to the PS may play a role in regulating ovulation intervals. Lastly, T_3_ and E_2_ treatment in vitro inferred that LEPH and HEPH respond differently to thyroid hormone and estradiol feedback on the pituitary gland.

## Methods

### Hen selection and tissue collection

All animal procedures were approved by the Institutional Animal Care and Use Committees at USDA Beltsville Agricultural Research Center (**BARC**) and at the University of Maryland, College Park (reference numbers 16–002 and XR-16-09, respectively). A total of 400 turkey hens over two equal sized flocks from the same commercial line were provided by a poultry primary breeding company (Hendrix Genetics, Kitchener, Ontario) and housed at BARC individually in wire cages during two separate time periods 6 months apart (200 hens per flock). Hens were managed with artificial lighting (14 L:10D) and were provided feed and water ad libitum to NRC standards (diet composition in Supplemental File 4). Daily egg records were kept from the onset of lay (around 28 weeks of age) until sampling occurred (at 37 weeks of age). Daily egg records were used to identify the bottom and top 15% of egg production for classification into two groups, LEPH and HEPH, as previously described [6]. Daily egg records were also used to predict the timing of the PS prior to ovulation, as described previously [15]. Average egg production and distribution for LEPH and HEPH groups did not differ between the two flocks used.

All hens were euthanized by cervical dislocation prior to tissue isolation. Additionally, all hens were sampled on the second day of the hen’s sequence. From the LEPH and HEPH groups, a total of 12 hens were sampled from the first flock and 6 hens were sampled from the second flock, based on the progression of the hen’s sequence and the timing of the preovulatory surge. Experimental replicate numbers per group were determined through a power analysis (α = 0.05, power = 0.8, |μ1 − μ2| = 0.5, σ2 = 0.2), with a recommended sample size of three replicates per group. The first flock (12 hens sampled total) was used to perform transcriptome analysis of the hypothalamus and pituitary, with six LEPH and six HEPH, half sampled outside of the PS and half sampled during the PS (*n* = 3 per group). These samples were also used to confirm gene expression results obtained from RNA sequencing through RT-qPCR (n = 3 per group). For this experiment, the hypothalamus and pituitary were isolated from each hen, snap frozen as whole tissues in liquid nitrogen, and stored at − 80 °C prior to assessment through RNA sequencing and confirmation of RNA sequencing results as described below. The second flock (6 hens sampled total) was used to perform follow up in vitro pituitary cultures, with three LEPH and three HEPH sampled exclusively outside of the PS (n = 3 per group). For the follow-up experiment, the pituitary was isolated from each hen and placed in ice cold Dulbecco’s Modified Eagle Medium (DMEM) until dispersion, cell culture, and RT-qPCR as described below. For both experiments, blood samples were taken from the wing vein immediately before sampling to measure plasma progesterone levels to confirm correct sampling outside or during the PS. Blood samples were collected and fractionated as previously described [7]. Plasma samples were stored at − 20 °C prior to assessment through radioimmunoassays as described below.

### Radioimmunoassay

Plasma progesterone levels were measured using a coated tube radioimmunoassay (**RIA**) kit (MP Biomedicals, Solon, OH) to confirm that sampling occurred at the correct time during the ovulatory cycle, based on experimental group assigned. All protocols were performed as directed by the supplier and samples were assayed in duplicate in a single RIA. Ether extraction of plasma samples prior to progesterone assessment and standard curve assessment were performed as previously described [6]. Hypothalamus and pituitary samples taken from a hen with plasma progesterone levels less than 1 ng/dL were considered to be sampled outside of the PS, while hypothalamus and pituitary samples taken from a hen with plasma progesterone levels greater than 4 ng/dL were considered to be sampled during of the PS. The plasma progesterone cutoffs for outside and during the PS were based on previous studies determining average plasma progesterone levels during the ovulatory cycle [15].

### RNA isolation, cDNA library construction, and sequencing

Total RNA was isolated from whole tissue hypothalamus and pituitary samples with RNeasy Mini kits (Qiagen, Valencia, CA), including on-column deoxyribonuclease digestion. Quantification of RNA was performed as previously described [15]. Amplified cDNA was generated using a SMART-Seq v4 Ultra Low Input RNA kit (Takara Bio Inc., Kusatsu, Japan) following the manufacturer’s procedure, starting with 10 ng of total RNA per sample. cDNA was amplified by long distance PCR (LD PCR) (8 cycles as per the manufacturer recommendation for 10 ng of starting RNA). Amplified cDNA was purified using Agencourt AMPure XP kit (Beckman Coulter, Indianapolis, IN). Amplified cDNA was quantified using an Agilent 2100 Bioanalyzer and High Sensitivity DNA Kit (Agilent, Santa Clara, CA).

Sequencing libraries were generated using a Nextera XT DNA Library Prep kit (Illumina, San Diego, CA) with input of 150 pg of amplified cDNA per library, following the manufacturer’s procedure. For each sample, two libraries were produced (from the same amplified cDNA), with a unique index pairing for library. Libraries were purified using Agencourt AMPure XP kit (Beckman Coulter, Indianapolis, IN) and were quantified using an Agilent 2100 Bioanalyzer and High Sensitivity DNA Kit. For sequencing 24 libraries (2 tissues) were pooled (10 nM). Libraries were pooled so that set 1 for each tissue was sequenced in a different pool than set 2. Pools were submitted to NC State’s GSL facility for paired-end sequencing (75 bp reads) on an Illumina NextSeq 500.

### Bioinformatic analysis of sequencing data

All FASTQ sequencing files have been deposited to the NIH Short Read Archive (accession numbers SAMN11624488-SAMN11624511). Processing and analysis of sequencing data was performed using the Galaxy (https://usegalaxy.org/) suite. Adapter sequences and low-quality sequences (Phred < 20) were removed from FASTQ files using the TrimGalore tool. Trimmed reads were mapped to the *Meleagris gallopavo* reference genome (Turkey_2.01 using ENSEMBL annotation release 98; https://uswest.ensembl.org/Meleagris_gallopavo/Info/Index). TopHat was used to analyze mRNA libraries. DEGs were determined using the Cuffdiff tool. Pairwise comparisons were made between LEPH and HEPH for each timepoint in the ovulatory cycle as well as between timepoints in the ovulatory for each egg production group. Due to poor annotation of the turkey genome, the protein sequences for DEGs that were unannotated in the turkey were subjected to orthologous comparisons in human, mouse, and chicken protein sequences using Ensembl Biomart (https://useast.ensembl.org/info/data/biomart/index.html). Unannotated DEGs were assumed orthologous if greater than 50% identity to the human, mouse, and chicken was seen at the protein level.

### Ingenuity pathway analysis

Ingenuity pathway analysis (**IPA**) (Qiagen, Valencia, CA) was performed on the differential expression data. IPA was used to construct gene networks as well as to predict upstream biological regulators for each pairwise comparison [33]. Only DEGs with RPKM> 0.2 were used for IPA. The RPKM threshold was selected based on the distribution of log_2_ transformed RPKM values across all of the comparisons examined. The threshold of DEGs was set at *P* < 0.05 and absolute fold change ≥1.5. Pathways and predicted upstream regulators with *P*-value < 0.05 (Fischer’s exact test) were considered to be statistically significant. For upstream regulators, published findings in the Ingenuity knowledge database were used to calculate the activation z-score to infer activation or inhibition of transcriptional regulators. Upstream regulators with a z-score greater than 2 or less than − 2 and P < 0.05 were considered to be significantly activated or inhibited. Networks and legends derived from Ingenuity Pathway Analysis are subject to copyright owned by Qiagen Inc.

### Culture of pituitary cells

All cell isolation procedures were performed using Minimum Essential Medium, Spinner modification (**SMEM**) or DMEM as noted below. Media supplementation, dispersion of pituitary cells, and plating of pituitary cells were performed as previously described [7]. Pituitary cells were pretreated with either no pretreatment (**NPT**) (10 μl DMEM/F12 added), T_3_ (1.5 ng/mL of thyroid hormone), or E_2_ (1.5 ng/mL of estradiol) for 12 h, followed by treatment with chicken GnRH-I (Phoenix Pharmaceuticals, Burlingame, CA) at 0, 10^− 9^, or 10^− 8^ M for 6 h (*n* = 3 per pretreatment, treatment, and egg production level combination). Incubation settings, cell retrypsinization, and cell storage were performed as previously described [7].

RNA isolation, quantification of RNA, RT, RT-qPCR, and primer design were performed as previously described [15]. Pituitary data normalization and analysis were performed as previously described [7]. Data are presented as fold increase over levels in LEPH basal cells for each pretreatment/treatment combination and time point.

### Confirmation of gene expression as determined by RNA sequencing

RNA extracted and quantified from whole tissue hypothalamus and pituitary samples for RNAseq was reverse transcribed as described previously [15]. PCR reactions and associated data analysis were performed as previously described [7]. For each tissue, mRNA levels for 12 genes total were determined. DEGs selected for RNAseq confirmation fit the following parameters: *P* < 0.05, absolute fold change greater or equal to 1.5, annotated in the turkey genome, and encoded by a single transcript. DEGs fitting these parameters were selected with the following RNAseq expression profiles: 3 DEGs up-regulated in LEPH both outside and during the PS, 3 DEGs up-regulated in HEPH both outside and during the PS, 3 DEGs which showed up-regulation in one egg production group outside of the PS and up-regulation in the other egg production group during the PS, and 3 control genes which did not show expression changes between egg production levels or during the ovulatory cycle. Primers were designed as described above. Data are presented as fold increase over mRNA levels for LEPH outside of the PS for each gene.

### Statistics

SAS software (SAS Institute, Cary, NC) was used to analyzed all log_2_ transformed gene expression data. For the pituitary cell culture data and RNAseq confirmation data, the mixed models procedure (PROC MIXED) was used to conduct a three-way ANOVA and a two-way ANOVA, respectively. The test of least significant difference (PDIFF statement) was used to compare the least squares means for each group when an overall significance level of *P* < 0.05 was indicated.

## Supplementary information

**Additional file 1: Figure S1.** Transcriptome alignment and mapping. (A) The number of reads obtained for each sample. The portion of mapped reads for each sample is represented in blue, whereas the portion of unmapped reads for each sample is represented in orange (mapped to Turkey 2.01 using ENSEMBL annotation release 98). (B) The number of aligned pairs obtained for each sample. The portion of aligned pairs with proper alignment is represented in blue, with discordant alignment in orange, and with multiple alignments in gray (mapped to Turkey 2.01 using ENSEMBL annotation release 98). **Figure S2.** Hypothalamic network analysis. Ingenuity® Pathway Analysis (Qiagen, Valencia, CA) was used to generate networks to biologically interpret the expression data. Copyright permission has been obtained from QIAGEN for use of the images presented. (A) Hypothalamic network comparing low egg producing hen (LEPH) and high egg producing hens (HEPH) gene expression during the preovulatory surge (PS) (RPKM> 0.2, *P* < 0.05, |fold change| > 1.5). Green represents genes up-regulated in LEPH, whereas red represents genes up-regulated in HEPH. (B) Hypothalamic network comparing LEPH and HEPH gene expression during the PS (RPKM> 0.2, P < 0.05, |fold change| > 1.5). Green represents genes up-regulated in LEPH, whereas red represents genes up-regulated in HEPH. **Figure S3.** Pituitary network analysis. Ingenuity® Pathway Analysis (Qiagen, Valencia, CA) was used to generate networks to biologically interpret the expression data. Copyright permission has been obtained from QIAGEN for use of the images presented. (A) Pituitary network comparing low egg producing hen (LEPH) and high egg producing hens (HEPH) gene expression during the preovulatory surge (PS) (RPKM> 0.2, P < 0.05, |fold change| > 1.5). Green represents genes up-regulated in LEPH, whereas red represents genes up-regulated in HEPH. (B) Pituitary network comparing HEPH gene expression outside and during the PS (RPKM> 0.2, P < 0.05, |fold change| > 1.5). Green represents genes up-regulated outside of the PS, whereas red represents genes up-regulated during the PS.

**Additional file 2:.** Supplement tables

**Additional file 3:.** Supplemental file 1

**Additional file 4:.** Supplemental file 2

**Additional file 5:.** Supplemental file 3

**Additional file 6:.** Supplemental file 4

## Data Availability

The FASTQ sequencing file datasets supporting the results of this article are available in the NCBI Short Read Archive (SRA; https://www.ncbi.nlm.nih.gov/sra), accession numbers, SAMN11624488-SAMN11624511. Differential expression output generated or analyzed during this study are included in this published article (Supplemental Files 1 and 2). Pathway analysis output generated during this study are included in this published article (Supplemental File 3). The genome assembly used in this study is available through Ensembl (Turkey_2.01; https://uswest.ensembl.org/Meleagris_gallopavo/Info/Index).

## Abbreviations

*ACTA2*: Alpha-actin-2
*ALB*: Albumin
*AR*: Androgen receptor
BARC: Beltsville Agricultural Research Center
*BLOC1S4*: Biogenesis of lysosomal organelles complex-1, subunit 4
*CGA*: Glycoprotein hormones alpha subunit
*COX2*: Cyclooxygenase-2
*CRYBB1*: Crystallin beta B1
*CSNK2A2*: Casein kinase 2 alpha 2
*CYP19A1*: Aromatase
DEGs: Differentially expressed genes
*DIO1*: Iodothyronine deiodinase 1
DMEM: Dulbecco’s modified eagle medium
E_2_: Estradiol pretreatment
EPD: Eggs per day
*ESRRB*: Estrogen related receptor beta
*ESYT3*: Extended synaptotagmin 3
*FA2H*: Fatty acid 2-hydroxylase
*FABP6*: Fatty acid binding protein 6
FSH: Follicle stimulating hormone
*FSHB*: Follicle stimulating hormone beta subunit
GnIH: Gonadotropin inhibitory hormone
*GNIH*: Gonadotropin inhibitory hormone
GnRH: Gonadotropin releasing hormone
*GNRH1*: Gonadotropin releasing hormone 1
*GNRHR*: Gonadotropin releasing hormone receptor
HEPH: High egg producing turkey hen
HPG: Hypothalamo-pituitary-gonadal
HPT: Hypothalamo-pituitary-thyroid
*HSD17B2*: Hydroxysteroid 17 beta dehydrogenase 2
*IGF1*: Insulin like growth factor 1
IPA: Ingenuity® Pathway Analysis
*ITG2A*: Integral membrane protein 2A
*ITGAV*: Integrin, alpha V subuit
*ITGB3*: Integrin, beta 3 subuit
LEPH: Low egg producing turkey hen
LH: Luteinizing hormone
*LHB*: Luteinizing hormone beta-subunit
*LYVE1*: Lymphatic vessel endothelial hyaluronan receptor 1
*MANEA*: Mannosidase endo-alpha
*ND4*: NADH dehydrogenase 4
NPT: No pretreatment
*NPVF*: Neuropeptide VF precursor
*PAPPA2*: Pappalysin 2
*PGK1*: Phosphoglycerate kinase 1
*PRL*: Prolactin
*PRLR*: Prolactin receptor
*PRPF19*: Pre-MRNA processing factor 19
PS: Preovulatory surge
*PSMA2*: Proteasome subunit alpha 2
*PSMD2*: Proteasome 26S subunit, non-ATPase 2
RIA: Radioimmunoassay
*RSAD2*: Radical s-adenosyl methionine domain containing 2
*SLC16A12*: Solute carrier family 16 member 12
*SLC7A5*: Solute carrier family 7 member 5
*SLCO1C1*: Solute carrier organic anion transporter family member 1C1
SMEM: Minimum essential medium, spinner modification
*SST*: Somatostatin
*STAR*: Steroidogenic acute regulatory protein
T_3_: Thyroid hormone pretreatment
*TGFBR1*: Transforming growth factor beta receptor 1
*TRHR*: Thyroid releasing hormone receptor
TSH: Thyroid stimulating hormone
*TSHB*: Thyroid stimulating hormone beta-subunit
*TSHR*: Thyroid stimulating hormone receptor
*TTR*: Transthyretin
*ZFPM1*: Zinc finger protein, FOG family member 1
