# Supplementary figures and images for: Transcriptome analysis of the hypothalamus and pituitary of turkey hens with low and high egg production
Source: BMC Genomics. 2020 Sep 21;21:647. doi: 10.1186/s12864-020-07075-y (PMC7507666; doi:10.1186/s12864-020-07075-y)

A

## Total Reads

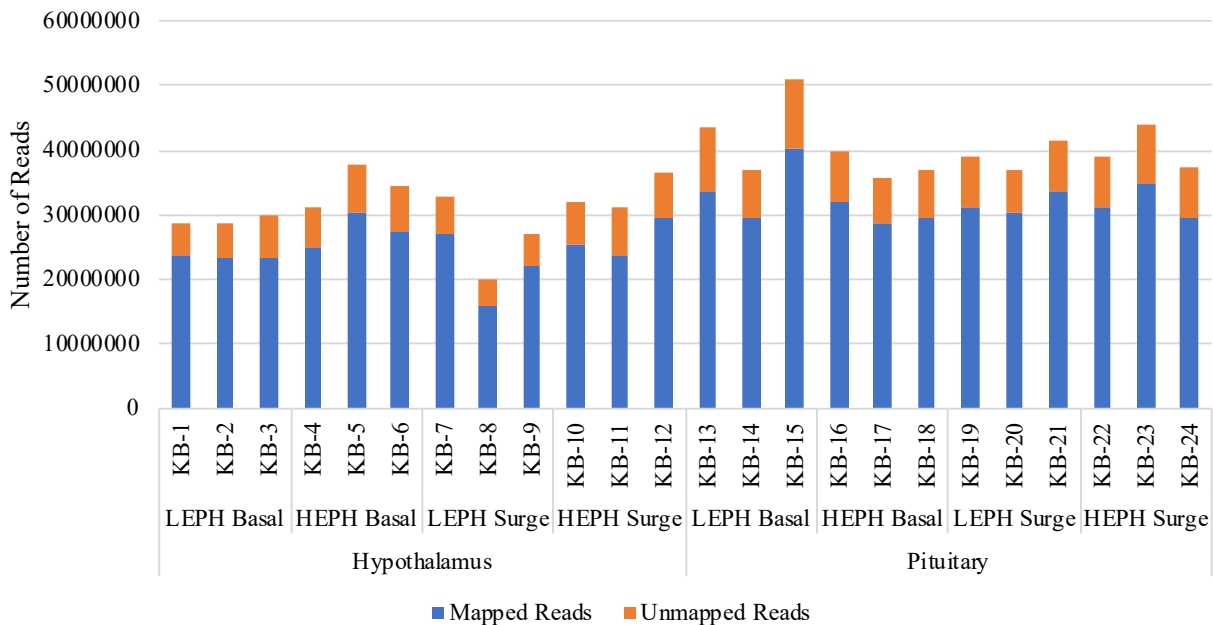

B

## Aligned Pairs

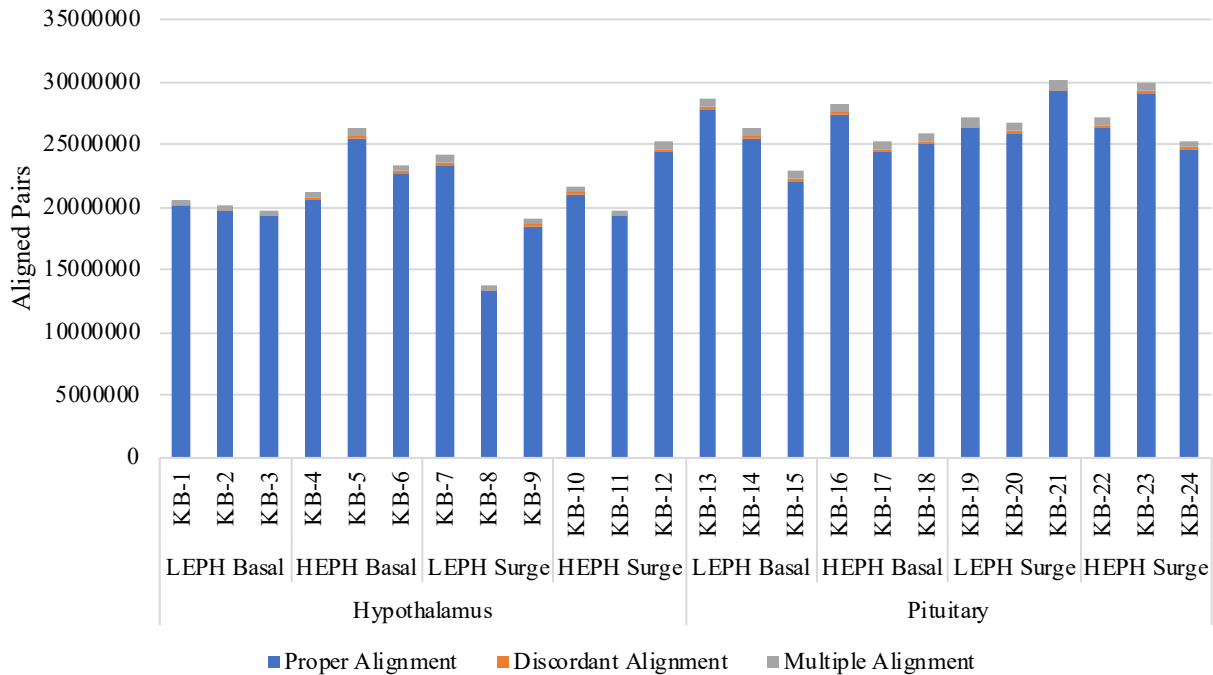

A

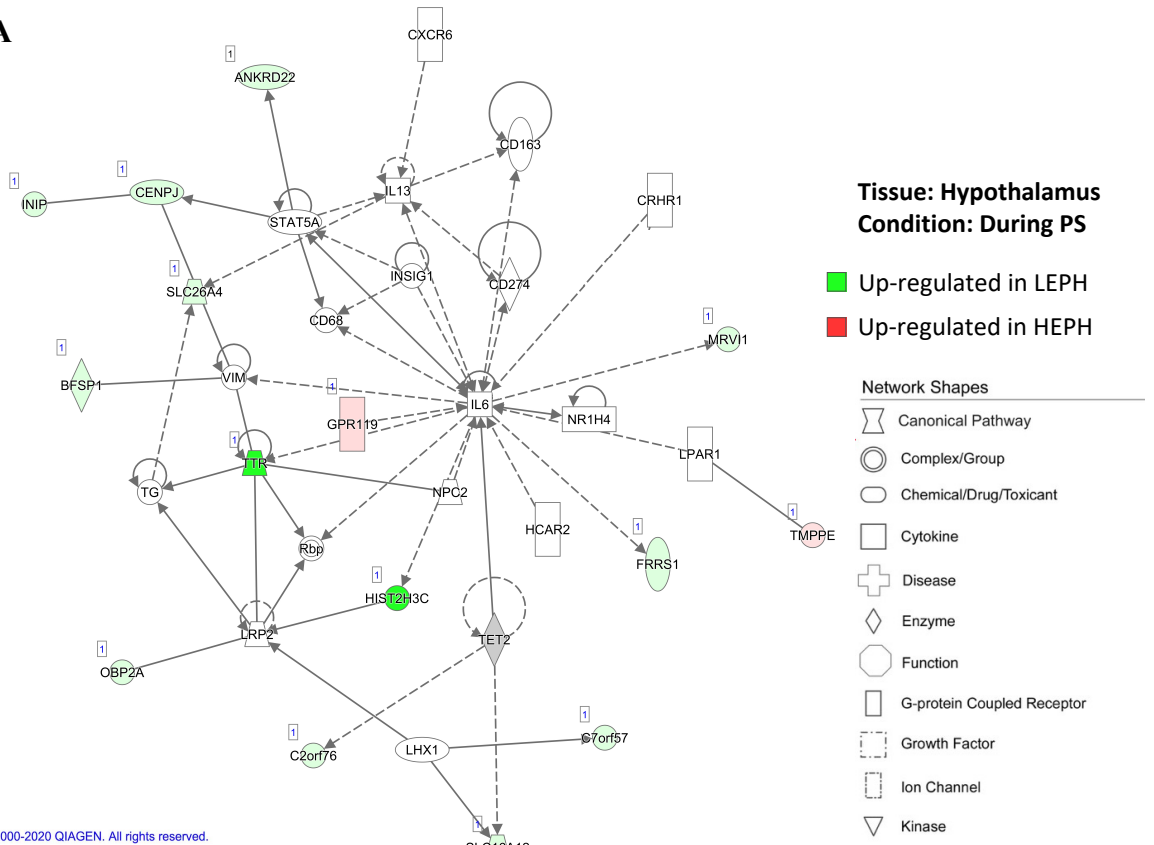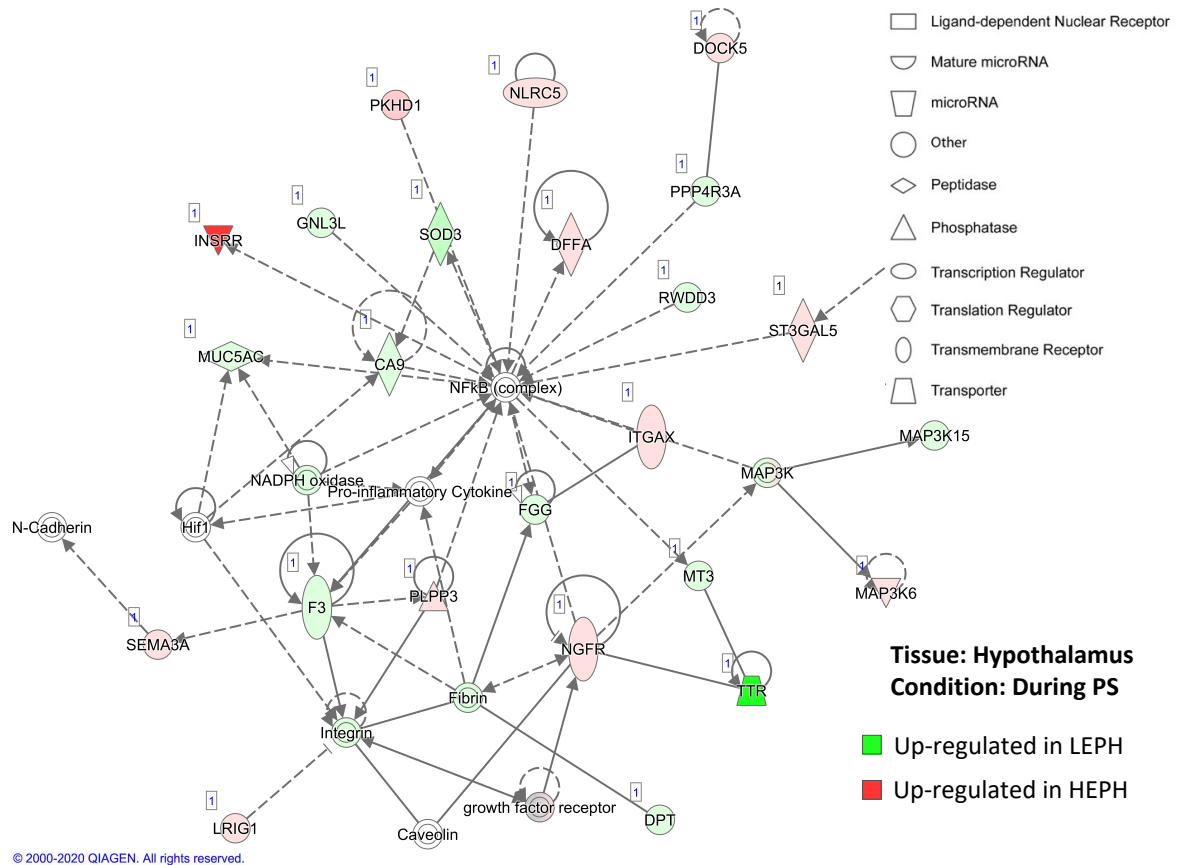

**A**

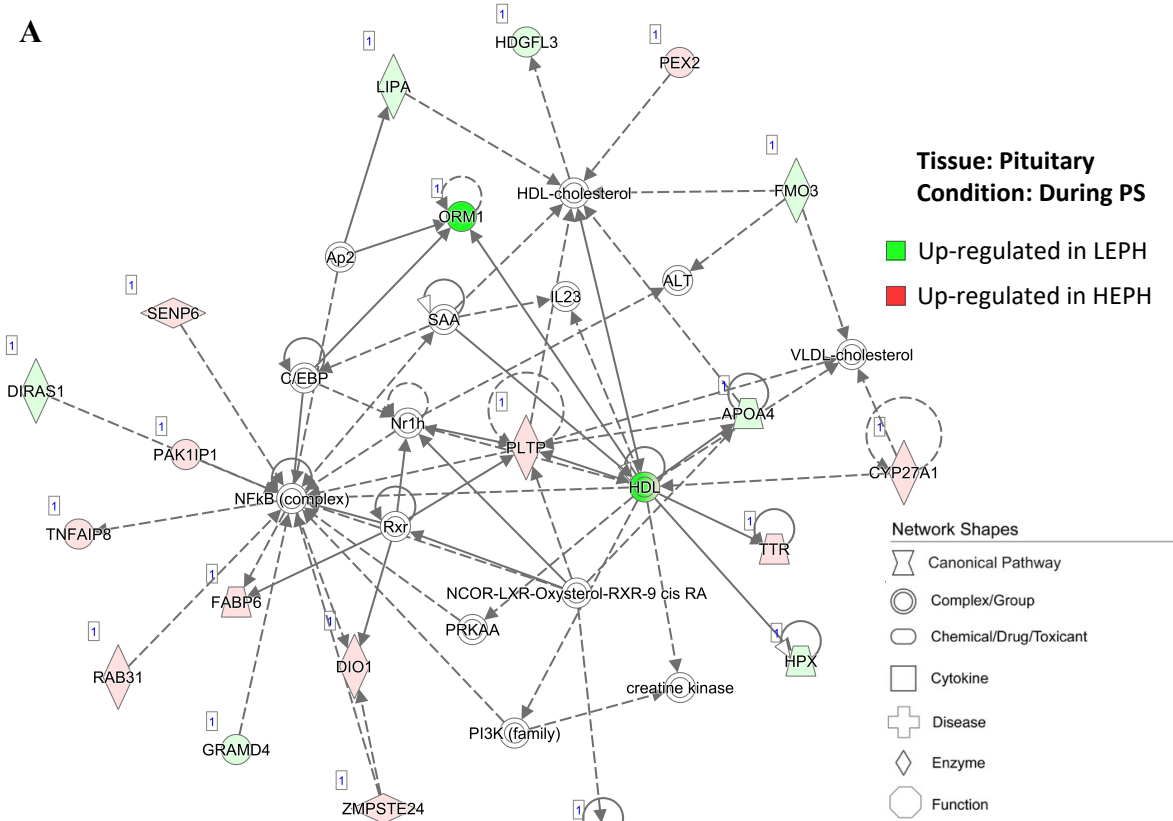

© 2000-2020 QIAGEN. All rights reserved.

**B**

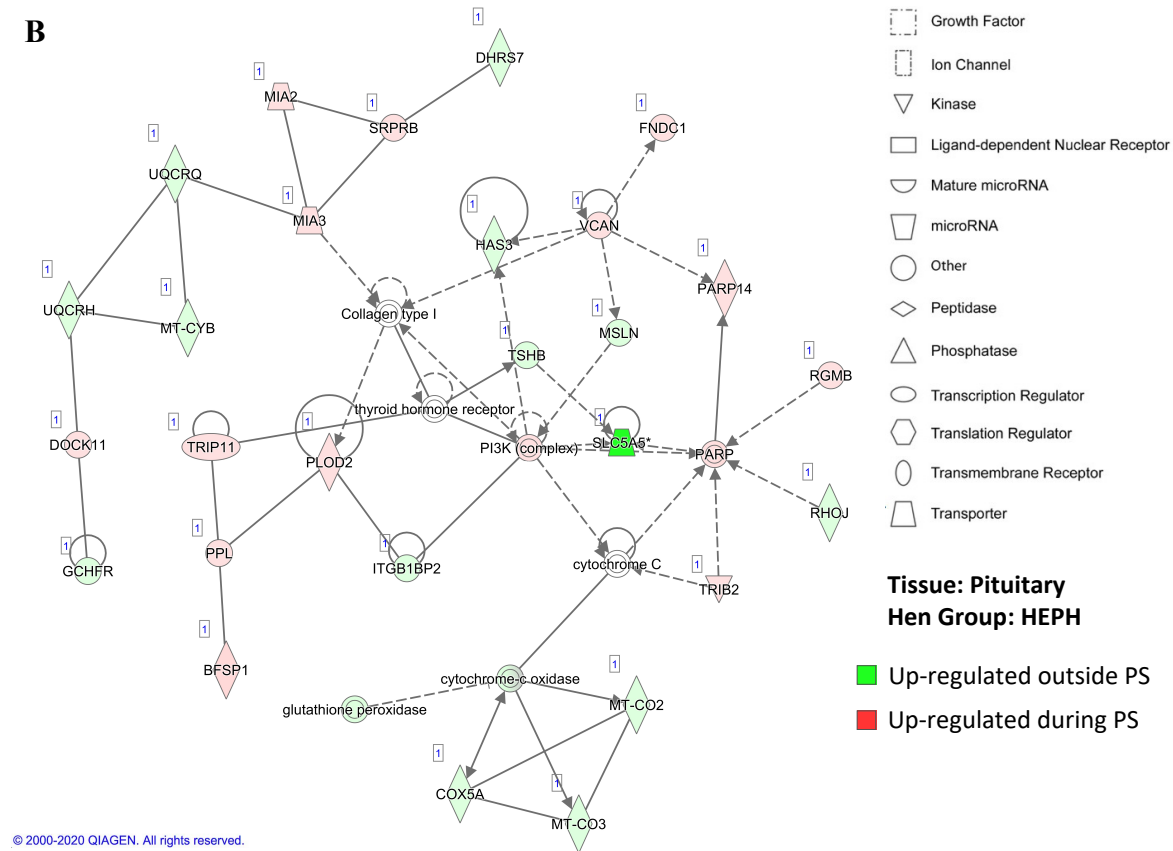

© 2000-2020 QIAGEN. All rights reserved.

Supplement: Supplementary file 1 — Additional file 1: Figure S1. Transcriptome alignment and mapping. (A) The number of reads obtained for each sample. The portion of mapped reads for each sample is represented in blue, whereas the portion of unmapped reads for each sample is represented in orange (mapped to Turkey 2.01 using ENSEMBL annotation release 98). (B) The number of aligned pairs obtained for each sample. The portion of aligned pairs with proper alignment is represented in blue, with discordant alignment in orange, and with multiple alignments in gray (mapped to Turkey 2.01 using ENSEMBL annotation release 98). Figure S2. Hypothalamic network analysis. Ingenuity® Pathway Analysis (Qiagen, Valencia, CA) was used to generate networks to biologically interpret the expression data. Copyright permission has been obtained from QIAGEN for use of the images presented. (A) Hypothalamic network comparing low egg producing hen (LEPH) and high egg producing hens (HEPH) gene expression during the preovulatory surge (PS) (RPKM> 0.2, P < 0.05, |fold change| > 1.5). Green represents genes up-regulated in LEPH, whereas red represents genes up-regulated in HEPH. (B) Hypothalamic network comparing LEPH and HEPH gene expression during the PS (RPKM> 0.2, P < 0.05, |fold change| > 1.5). Green represents genes up-regulated in LEPH, whereas red represents genes up-regulated in HEPH. Figure S3. Pituitary network analysis. Ingenuity® Pathway Analysis (Qiagen, Valencia, CA) was used to generate networks to biologically interpret the expression data. Copyright permission has been obtained from QIAGEN for use of the images presented. (A) Pituitary network comparing low egg producing hen (LEPH) and high egg producing hens (HEPH) gene expression during the preovulatory surge (PS) (RPKM> 0.2, P < 0.05, |fold change| > 1.5). Green represents genes up-regulated in LEPH, whereas red represents genes up-regulated in HEPH. (B) Pituitary network comparing HEPH gene expression outside and during the PS (RPKM> 0.2, P < 0.05, |fold [file 12864_2020_7075_MOESM1_ESM.pdf]
