# Supplementary material for: Transcriptome analysis of the hypothalamus and pituitary of turkey hens with low and high egg production
Source: BMC Genomics. 2020 Sep 21;21:647. doi: 10.1186/s12864-020-07075-y (PMC7507666; doi:10.1186/s12864-020-07075-y)
Supplement: Supplementary file 2 — Additional file 2:. Supplement tables [file 12864_2020_7075_MOESM2_ESM.docx]

**Table S1**. **Significant gene expression changes in the hypothalamo-pituitary-gonadal (HPG) axis during the preovulatory surge**. Fold change during the preovulatory surge and significance are presented for key HPG axis genes in low egg producing hens (LEPH) and high egg producing hens (HEPH) (RPKM>0.2, P<0.05).

| **LEPH** | | | | |
| --- | --- | --- | --- | --- |
| Tissue | Gene | Function | Fold Change | P-Value |
| Hypothalamus | NPVF | HPG axis signaling | 1.38 | 0.0470 |
|  | FSHR | HPG axis signaling | 4.85 | 0.0033 |
|  | HSD17B1 | steroid hormone biosynthesis | 9.63 | 0.0358 |
|  | HSD11B1 | steroid hormone biosynthesis | 1.66 | 0.0341 |
| Pituitary | GNRHR | HPG axis signaling | -1.72 | 0.0273 |
|  | PRLR | prolactin signaling | 1.65 | 0.0008 |
|  | STAR | steroid hormone biosynthesis | -9.64 | 9.11E-07 |
|  | BMP6 | steroid hormone biosynthesis | -1.71 | 0.0240 |
| **HEPH** | | | | |
| Tissue | Gene | Function | Fold Change | P-Value |
| Hypothalamus | GNRH1 | HPG axis signaling | -3.63 | 0.0005 |
|  | AR | steroid hormone signaling | 1.56 | 0.0374 |
|  | PRL | prolactin signaling | 2.34 | 0.0118 |
| Pituitary | NPFFR2 | HPG axis signaling | 2.71 | 0.0428 |
|  | GNRHR | HPG axis signaling | -1.78 | 0.0496 |
|  | CGA | HPG axis signaling | -1.63 | 0.0139 |

**Table S2.** **Significant gene expression changes in the hypothalamo-pituitary-thyroid (HPT) axis during the preovulatory surge**. Fold change during the preovulatory surge and significance are presented for key HPT axis genes in low egg producing hens (LEPH) and high egg producing hens (HEPH) (RPKM>0.2, P<0.05).

| **LEPH** | | | | | |
| --- | --- | --- | --- | --- | --- |
| Tissue | Gene | Function | Fold | P-Value |  |
| Hypothalamus | TRHR | HPT axis signaling | -1.71 | 0.0041 |  |
|  | TTR | thyroid hormone transporter | 61.89 | 4.76E-14 |  |
|  | SLC16A1 | thyroid hormone transporter | 1.35 | 0.0101 |  |
| Pituitary | TRHR | HPT axis signaling | 1.42 | 0.0123 |  |
|  | ATP1B4 | thyroid hormone synthesis | -2.68 | 0.0088 |  |
|  | SLC26A4 | thyroid hormone synthesis | 3.19 | 4.49E-05 |  |
|  | TTR | thyroid hormone transporter | 4.56 | 0.0118 |  |
|  | ALB | thyroid hormone transporter | 5.57 | 0.0163 |  |
|  | SLC7A5 | thyroid hormone transporter | -1.40 | 0.0380 |  |
| **HEPH** | | | | | |
| Tissue | Gene | Function | Fold | P-Value |  |
| Hypothalamus | THRA | thyroid hormone receptor | 2.13 | 0.0424 |  |
|  | TTR | thyroid hormone transporter | -4.40 | 0.0049 |  |
|  | SLC16A10 | thyroid hormone transporter | -1.56 | 0.0226 |  |
| Pituitary | TSHB | HPT axis signaling | -1.94 | 0.0210 |  |
|  | CGA | HPT axis signaling | -1.63 | 0.0139 |  |
|  | SLC5A5 | thyroid hormone synthesis | -23.57 | 0.0002 |  |

**Table S3.** **Predicted target genes of beta-estradiol differentially expressed between egg production levels**. Differentially expressed genes (DEGs) between low egg producing hens (LEPH) and high egg producing hens (HEPH), outside and during the preovulatory surge, that are predicted to be target genes of beta-estradiol (RPKM>0.2, P<0.05, |fold change|>1.5).

| **Tissue** | **Condition** | **Differentially Expressed Target Genes of Beta-Estradiol between LEPH and HEPH** |
| --- | --- | --- |
| Hypothalamus | Outside PS | ACTA2, ACTC1, ADORA2A, CD74, CREB1, CRLF1, CYBB, CYP19A1, GABRP, HSD17B2, MB, MGLL, MPEG1, OXTR, PRL, SMPDL3A, SRC, TLR2, TRHR, TSHB, TTR |
|  | During PS | ACKR3, ACTA2, ADGRG2, ASB9, ATAD2, CAD, CCNA1, COL1A2, CRIM1, DNMT3B, F3, FABP4, FHL1, GNRH1, GRIK4, KIF3C, KRT19, LYZ, MAP3K8, MMP9, MPEG1, NPVF, PAPSS2, PLEKHA6, PRL, RAMP3, SEMA3A, SHISA2, SLC6A20, SOCS2, SRC, SSTR3, THSD4, TNNC2, TPD52L1, TPH2, TSHR, TTR, USP53 |
| Pituitary | Outside PS | ADCY9, ADGRG2, ADRB1, ADRB3, ANK1, ANXA1, AP1B1, APOA4, AR, ARMT1, ATRX, BAG1, BLOC1S6, BNIP2, BRCA2, CAD, CASP3, CCNE1, CDC45, CDKL2, CGA, CSF3R, CTNND1, CUL4A, CXCL12, CYP26B1, CYTH3, DERL1, DNAJC3, DRD2, DUSP4, EIF3J, ERH, F2RL1, F3, FAS, FEZ2, FHL2, FN1, GALNT1, GCH1, GDNF, GHRHR, GHSR, GREB1, GRIK4, GUCY1A1, GYG1, HADH, HAPLN2, HSP90B1, IL17RD, IL18, INPP5J, KCNAB1, KCNN2, KIF3C, KPNA4, KRAS, LARP6, LRP8, MAL2, MAPK8IP1, MCM5, MMP13, MMP9, MTMR4, MXD1, MYLK, NCOA1, NET1, NRIP3, NRP2, NUCB2, OGDH, OGN, PAK5, PAPSS2, PFKL, PGRMC1, PI4KB, PLEKHA6, PMM2, POLA1, PPARG, PPL, PPRC1, PRKCH, PROS1, PSAT1, PSD, PSMA2, PTPRN, PTPRU, RAB9A, RALA, RER1, RHOQ, RMND1, ROCK1, SEZ6, SLC2A1, SLC38A2, SLC6A20, SLC7A5, SNED1, SRP54, SSR1, STAR, SUCLA2, TANK, TNFSF13B, TRAPPC2B, TRNT1, TUFT1, UBE2B, UBL3, USP19, USP53, YWHAZ |
|  | During PS | ACTA2, APOA1, APOA4, ARC, ATF3, ATP2B1, ATRX, BAG1, BMPR2, C3, CCDC170, CCNA1, CRLF1, CYP26B1, CYTH3, DNAJC3, EDN2, FHL1, FMR1, GALNT1, GJB1, HAS2, HSP90B1, HSPA4, HTR2C, IGFBP2, IL17RD, LUM, MGP, MMP9, MMRN1, MYOF, NELL2, NUCB2, PADI1, PMM2, RAB31, RALA, RGS3, RP2, SNCG, SST, TRIB2, TRNT1, TSHB, TTR, UBL3, USP8, VAV3 |

**Table S4.** **Predicted target genes of beta-estradiol differentially expressed during the ovulatory cycle.** Differentially expressed genes during the ovulatory cycle individually in low egg producing hens (LEPH) and high egg producing hens (HEPH), that are predicted to be target genes of beta-estradiol (RPKM>0.2, P<0.05, |fold change|>1.5).

| **Tissue** | **Group** | **Differentially Expressed Target Genes of Beta-Estradiol Outside and During the PS** |
| --- | --- | --- |
| Hypothalamus | LEPH | ACKR3, ACTA2, ATAD2, CAD, CHAT, CHRM3, CRIM1, EGFR, F2RL1, FADD, FGD6, FOXN3, GABRP, HSD11B2, KITLG, KRT19, LEPR, MMP9, POLA1, PTGS2, RASGRP1, RUNX2, SEMA3A, SEMA3C, SLC12A4, SLC6A20, SLIT2, SMPDL3A, SP4, SRC, SST, TACSTD2, THSD4, TRHR, TTR, USP53 |
|  | HEPH | ABCA1, ACTA2, ACTC1, AR, ARC, AURKA, EDN2, GNRH1, HSD11B2, MATN2, NR5A2, ORC1, PDYN, PLEKHA6, PPP1R1B, PRL, RASGRP1, RNF4, SRC, TNNC2, TPD52L1, TTR, VAV3 |
| Pituitary | LEPH | ACTA2, ACTC1, ADAMTS5, ADGRG2, ADRB1, ALB, ARMT1, ASB9, BAG1, BRCA2, CCNE1, CDKL2, CLCA2, CSF3R, CYTH3, DDC, F3, FABP3, FAS, FBN1, FMOD, GALR1, GHSR, GPR12, GUCY1A1, HSD11B2, HTR2C, IL17RD, IL18, IP6K3, KCND3, KCNN2, KRAS, LRP8, MMP9, NRP1, PDK4, PMM2, POMC, PPARG, PPM1K, PREP, PRLR, PROM1, PSD, PTGS2, PTPRN, RMND1, SHISA2, SNCG, SPRY2, SRP54, SST, SSTR1, STAR, THSD4, TNNC2, TP53INP1, TSKU, TTR, TUFT1, UBE2B |
|  | HEPH | ACTC1, ADAMTS1, ADIPOQ, AGTR1, ANK1, APOA1, ARC, ATAD2, ATP5ME, CAMK2A, CGA, COX5A, CRABP2, CYP24A1, EGF, ELOVL2, EREG, ERH, FABP3, GCHFR, GREB1, GRHL2, GRIN2C, HBEGF, HSD11B2, HSPB1, IGFBP2, IL17RD, IL18, LGALS3, MGP, MMP9, MMRN1, MYC, NEDD9, NET1, NIPSNAP1, NPTX1, NRIP3, OPRK1, PAPSS2, PCBD1, PDK4, PDZK1IP1, PLEKHA6, PPL, PPM1K, PPP1R1B, RBPMS, RDH10, RNF4, RPL21, S100A6, SHISA2, SIN3B, SPRY2, SQSTM1, SRC, STC1, THSD4, TMEM258, TMOD2, TP53I11, TP53INP1, TPR, TRIB2, TSHB, UCK2, VAV3 |
