# Supplementary material for: Transcriptome analysis of the hypothalamus and pituitary of turkey hens with low and high egg production
Source: BMC Genomics. 2020 Sep 21;21:647. doi: 10.1186/s12864-020-07075-y (PMC7507666; doi:10.1186/s12864-020-07075-y)
Supplement: Supplementary file 6 — Additional file 6:. Supplemental file 4 [file 12864_2020_7075_MOESM6_ESM.pdf]

Turkey Breeder Diet		
**Ingredient**	**As Fed (lbs)**	**Dry Matter (lbs)**
Corn	2601	2223.855
48% Soybean Meal	706	706
Cal. Carb.	235.16	235.16
Alfalfa Meal 17%	175	161
Zinc Oxide	92.4	89.628
PRO LAK	70	65.135
Soy Oil	65	64.35
Turkey Vit.	20	20
SALT	12	12
Choline 60%	5.9	5.9
DL-Methionine	5.44	5.44
Lysine	5	5
Trace Min.	4	4
Selenium Premix	4	4

**Dry Matter**	90.02%

Estimated Daily Intakes	
AF Intake	1.11 lb/day
DM Intake	1 lb/day
